# Coalition of Oct4A and β1 integrins in facilitating metastasis in ovarian cancer

**DOI:** 10.1186/s12885-016-2458-z

**Published:** 2016-07-08

**Authors:** Chantel Samardzija, Rodney B. Luwor, Michael A. Quinn, George Kannourakis, Jock K. Findlay, Nuzhat Ahmed

**Affiliations:** Department of Obstetrics and Gynaecology, University of Melbourne, Parkville, Melbourne, VIC 3052 Australia; Department of Surgery, University of Melbourne, Parkville, Melbourne, VIC 3052 Australia; Fiona Elsey Cancer Research Institute, Suites 23-26, 106-110 Lydiard Street South, Ballarat Technology Central Park, Ballarat, 3353 Australia; Federation University Australia, Ballarat, VIC 3010 Australia; The Hudson Institute of Medical Research, Clayton, Melbourne, VIC 3168 Australia

**Keywords:** Ovarian carcinoma, Cancer stem cells, Metastasis, Integrins, Chemoresistance, Recurrence, Oct4A

## Abstract

**Background:**

Ovarian cancer is a metastatic disease and one of the leading causes of gynaecology malignancy-related deaths in women. Cancer stem cells (CSCs) are key contributors of cancer metastasis and relapse. Integrins are a family of cell surface receptors which allow interactions between cells and their surrounding microenvironment and play a fundamental role in promoting metastasis. This study investigates the molecular mechanism which associates CSCs and integrins in ovarian cancer metastasis.

**Methods:**

The expression of Oct4A in high-grade serous ovarian tumors and normal ovaries was determined by immunofluorescence analysis. The functional role of Oct4A was evaluated by generating stable knockdown (KD) of Oct4A clones in an established ovarian cancer cell line HEY using shRNA-mediated silencing. The expression of integrins in cell lines was evaluated by flow cytometry. Spheroid forming ability, adhesion and the activities of matrix metalloproteinases 9/2 (MMP-9/2) was measured by in vitro functional assays and gelatin zymography. These observations were further validated in in vivo mouse models using Balb/c nu/nu mice.

**Results:**

We report significantly elevated expression of Oct4A in high-grade serous ovarian tumors compared to normal ovarian tissues. The expression of Oct4A in ovarian cancer cell lines correlated with their CSC-related sphere forming abilities. The suppression of Oct4A in HEY cells resulted in a significant diminution of integrin β1 expression and associated α5 and α2 subunits compared to vector control cells. This was associated with a reduced adhesive ability on collagen and fibronectin and decreased secretion of pro-MMP2 in Oct4A KD cells compared to vector control cells. In vivo, Oct4A knock down (KD) cells produced tumors which were significantly smaller in size and weight compared to tumors derived from vector control cells. Immunohistochemical analyses of Oct4A KD tumor xenografts demonstrated a significant loss of cytokeratin 7 (CK7), Glut-1 as well as CD34 and CD31 compared to vector control cell-derived xenografts.

**Conclusion:**

The expression of Oct4A may be crucial to promote and sustain integrin-mediated extracellular matrix (ECM) remodeling requisite for tumor metastasis in ovarian cancer patients.

## Background

Ovarian cancer is a major gynaecological malignancy worldwide with 125,000 deaths reported each year [1]. The development of ascites and peritoneal metastases is a major clinical issue in the prognosis and management of ovarian cancer. A significant proportion of ovarian cancer cells within the peritoneal ascites exist as multicellular aggregates or spheroids which have the capacity to invade nearby organs [2]. The pathology of peritoneal-based metastasis includes the attachment of shed primary ovarian tumor cells onto the mesothelial-lined spaces of the peritoneum in the form of spheroids resulting in multiple tumor masses necessary for secondary growth. Current treatment strategies for advanced-stage ovarian cancer patients results in initial remission in up to 80 % of patients [3]. However, following a short remission period (usually 16–22 months), recurrence occurs in almost all patients ultimately resulting in patient mortality. This high rate of recurrence is largely due to the ability of tumor cells to evade the cytotoxic effects of chemotherapy associated with intrinsic or acquired chemoresistance, a property commonly associated with CSCs [4, 5].

The concept of CSCs supports the existence of a sub-population of tumor cells which drive tumor growth and progression, while also sustaining the cytotoxic pressure imposed by therapy to promote the re-growth of therapy-resistant tumors [6, 7]. In this scenario, it can be postulated that the development of an effective therapy for recurrent ovarian tumors will depend on the identification of tumor specific CSCs, as well as the pathways/regulators controlling their survival and sustenance.

Oct4 (Oct3/4 or POU5F1) is a member of the POU-domain family of transcription factors and has been shown to play an important role in the maintenance of self-renewal and pluripotency in embryonic stem cells (ESCs). It is commonly expressed in unfertilized oocytes, the inner cell mass (ICM) of a blastocyst, germ cells, embryonic carcinoma cells and embryonic germ cells [8]. Up regulation of Oct4 expression has been shown to sustain an undifferentiated pluripotent stem cell state, while a loss of Oct4 expression results in the induction of differentiation in stem cells, producing a heterogeneous population of highly specialized daughter cells [8]. Additionally, Oct4 has consistently been shown to be an integral factor necessary for the reprogramming of somatic cells into induced pluripotent stem cells (iPSCs). Although a cocktail of transcription factors are typically involved in this process (eg Oct4, Sox2, Klf4 and c-Myc), reprogramming efficiency is reduced if Oct4 is not present, thus indicating an absolute requirement for Oct4 in maintaining a stem cell-like state [9]. Importantly however, Oct4 is highly expressed in many tumor types, suggesting that the reprogramming of somatic cells as well as tumor development and progression may share common cellular mechanisms [10].

The Oct4 gene encodes for three isoforms, generated by alternative splicing of genes, known as Oct4A, Oct4B and Oct4B1 [11, 12]. At the nucleotide level, both Oct4A and Oct4B share exons 2–5. However, exon 1 is missing in Oct4B and is replaced by exon 2a [11, 12]. These differences appear to have significant biological implications on isoform function with Oct4A specifically expressed in the nucleus of ESCs, human somatic stem cells, tumor stem cells and in some adult stem cells [11, 12]. Oct4B on the other hand, is localised to the cytoplasm and expressed at low levels in human somatic stem cells, tumor cells, adult tissues as well as pluripotent stem cells. For investigations in stem cell biology, it is therefore crucial that the Oct4A isoform is specifically targeted.

The interaction between CSCs and the neighbouring microenvironment forms a ‘niche’ which is critical for sustaining the stemness of cancer cells [12]. Integrins are heterodimeric transmembrane receptors composed of a combination of different α and β subunits. They are essential in sensing the microenvironment and triggering cellular responses by bridging physical connections between the interior and exterior environments of cells [13]. This allows the flow of bi-directional signals that control basic cellular functions such as adhesion, migration, proliferation, and survival as well as differentiation [13]. In the context of CSCs, integrin receptors have been shown to promote a more malignant phenotype for tumor promotion and drug resistance [14, 15]. These receptors are highly expressed in stem cell niches and contribute to diverse CSC functions [14–16]. In this study using cancer cell lines, we demonstrate a direct link between the expression of α2, α5 and β1 integrin subunits with Oct4A expression in ovarian cancer and discuss the implications of these findings in relation to CSCs and progression of ovarian cancer in patients.

## Methods

### Patient samples

#### Tissue collection

Primary high grade serous epithelial ovarian tumors and normal ovarian tissues were obtained from patients requiring surgical resection at The Royal Women’s Hospital, Melbourne, Australia. The histopathological diagnosis, tumor grades and stages were determined by anatomical pathologists at the Royal Women’s Hospital as part of clinical diagnosis. Patients who were treated with chemotherapy prior to surgery were excluded from specimen collection. Tissues were paraffin embedded or snap frozen at the time of collection and stored at −80 °C until processed.

### Cell lines

Four established human epithelial ovarian cancer cell lines SKOV3, OVCAR5, OVCA433, and HEY were used in this study. The growth conditions of these cell lines have been described previously [17]. The human ovarian surface epithelial cell line (IOSE398) transfected with the SV-40 antigen was obtained from Dr Nelly Auersperg, University of British Columbia, Canada [18]. The development of the vector control, Oct4A KD1 and Oct4A KD2 cell lines and their growth conditions have been described previously [19]. Cells were routinely checked for mycoplasma infection.

### Antibodies

Mouse monoclonal anti-human Oct4A and Sox2 were obtained from R&D Systems (Minneapolis, Minnesota, USA) and Cell Signalling Technology (Danvers, Massachusetts, USA) respectively. Rabbit polyclonal anti-human GAPDH was obtained from IMGENIX (CA, USA). Mouse anti-human integrin α2 (CD49b), anti-human α5 integrin (CD49e) and anti-human β1 (CD29) were obtained from Millipore (Billerica, Massachusetts, USA). Goat F(ab')2 anti-mouse IgG was purchased from Southern Biotech (Birmingham, AL, USA). Rabbit polyclonal anti-human cytokeratin 7 (CK-7), anti-human Glut-1, anti-human CD34 and anti-human CD31 were obtained from Ventana (Tucson, USA). The DAPI nucleic acid stain and Alexa Fluor® 488 goat anti-mouse IgG were obtained from Life Technologies (Carlsbad, CA, USA). Ventana antibodies used for the immunohistochemical staining of tumor xenografts were obtained from Roche (Basel, Switzerland) as described previously [19–21].

### Immunofluorescence analysis

For primary tissue analysis, paraffin embedded tissue samples were sectioned at 5 μm and deparaffinised by xylene and graded ethanol wash. Slides were blocked for 10 min in CAS-Block™ Histochemical Reagent (Invitrogen Corporation). For non-adherent sphere populations, 100–200 μL of sphere containing media was added per chamber well containing 200 μL appropriate fresh growth media and cultured on 8 well μ-Slides (ibidi, Martinsried, Germany) for 24 h to allow for adhesion to plastic before being fixed with 4 % paraformaldehyde. Monolayer cell lines were seeded at 5 × 10^3^ cells per well onto the 8-well Nunc™ Lab-Tek™ Chamber Slide™ System (Thermo Scientific) and cultured as monolayer in complete RPMI-1640 growth media before being fixed with 4 % paraformaldehyde. Samples were probed overnight at 4 °C with either Oct4A (1:200), integrin β1 (1:200) or integrin α5 (1:200) primary antibodies, detected with Alexa Fluor® 488 Goat-Anti-Mouse antibody (1:200) and counterstained with 4’,6-diamidino-2-phenylindole (DAPI) (1:10,000). Fluorescence imaging was visualized and captured using an Olympus Cell^R^ fluorescence microscope and associated software (Olympus Corporation, Tokyo, Japan). Semi-quantitative analysis to assess fluorescence intensity of the antibody of interest was performed using the inbuilt Cell^R^ software. Results are expressed as a fold change of the protein of interest compared to DAPI for each analysis.

### RNA extraction and real-time PCR

Quantitative real-time PCR was performed as described previously [19]. Relative quantification of gene expression was normalized to 18S and calibrated to the appropriate control sample using the SYBR Green-based comparative CT method (2^-ΔΔCt^). The primer set of Oct4A and β1 integrin are described in Table 1. The probe for 18S has been described previously [22].

**Table 1:** Primer sequences of oligos used in quantitative Real-Time PCR

Oligo name	Forward (F) 5’-3’ Reverse (R) 5’-3’	Primer sequence
Oct4A	F	CTC CTG GAG GGC CAG GAAT C
	R	CCA CAT CGG CCTG TGT ATA T
Integrin β1	F	ATC CCA GAG GCT CCA AAG AT
	R	CTA AAT GGG CTG GTG CAG TT

### Western blotting

Cell lysates were extracted using the NU-PER nuclear and cytoplasmic extraction kit (Thermo Scientific, Waltham, MA, USA) as per manufacturer’s instructions. SDS-PAGE and Western blot was performed on the cell lysates as described previously [19].

### Sphere forming assay

The sphere forming ability of cells and subsequent sphere adhesion ability was determined as described previously [19]. Cellular aggregates with a diameter greater than 200 μm were classified as spheres.

### Flow cytometric analysis

Flow cytometry was used to assess the expression of cell surface makers as described previously [23]. Briefly, cells were grown as monolayer cultures, harvested and 10^6^ cells incubated with primary antibody (1:100) for 30 mins at 4 °C. Cells were washed with 1X PBS, stained with secondary Goat F(ab’)2 anti-mouse IgG antibody conjugated with phycoerythin for 30 mins at 4 °C and resuspended in 200 μL 1XPBS prior to flow cytometry analysis. All data was analysed using Cell Quest software (Becton-Dickinson, Bedford, MA, USA) and expressed as background IgG staining subtracted from the IgG staining of the antibody of interest.

### Adhesion assay

Cell adhesion assays were used to assess the ability of cells to adhere to extracellular matrix proteins. Briefly, 5 × 10^4^ cells were seeded in complete growth media on culture plates pre-coated with 10 μg/mL collagen, Type 1 (Sigma-Aldrich) or 10 μg/mL fibronectin (Sigma-Aldrich) with sterile 1X PBS used as a diluent. Cells were incubated for 90 mins at 37 °C in a humidified atmosphere in the presence of 5 % CO_2_. The growth media was removed and cells were washed vigorously with 1X PBS using an orbital rocker on full speed twice for 5 mins to remove non-adhering cells. Cells were fixed with 4 % paraformaldehyde before being stained for 10 mins with 5 % Crystal Violet (Sigma-Aldrich) diluted in 0.2 % ethanol. Following crystal violet staining, cells were gently rinsed with 1X PBS and plates allowed to dry at room temperature before performing a dry reading at OD_550nm_ with the SpectraMax190 Absorbance Microplate Reader and SoftMax® Pro Computer Software (Molecular Devices). Adhesion was calculated by subtracting the OD_550nm_ reading of the negative control from the OD_550nm_ reading of coated wells.

### Gelatin zymography

This was performed as described previously [24]. Briefly, complete growth medium from cells grown as sub-confluent monolayer cultures was discarded and replaced by serum free medium in a humidified atmosphere at 37 °C in the presence of 5 % CO_2_. After 48 h, the serum free medium was collected and concentrated using 10 kDa Amicon Ultra-4 spin columns (Merck-Millipore, Billerica, MA, USA). Samples were resolved on 10 % (v/v) Tris–HCl acrylamide gels containing 0.1 % (w/v) gelatin, washed and stained with 0.2 % Coomassie blue. The gel was de-stained and areas void of blue stain indicative of areas of enzyme activity. Semi-quantitative densitometric analysis was performed on all gels to determine the extent of enzymatic digestion using Image Quant software (GE Healthcare) and expressed as the intensity of Pro-MMP9 or Pro-MMP2 bands of interest.

### Animal studies

Animal experiments were performed on Balb/c nude mice as described previously [19–21, 25, 26].

### Immunohistochemistry of mouse tumors

Immunohistochemistry analysis of mouse tumors was performed as described previously [19–21, 25, 26].

### Statistical analysis

All results are presented as the mean ± standard error of the mean (SEM) of three independent experiments unless otherwise indicated. Statistical significance was measured compared to the vector control using one way-ANOVA and Dunnett’s Multiple Comparison test unless otherwise indicated. For primary tissue analysis, Student’s *t*-test was used to compare normal and high grade tissue samples. A probability level of <0.05 was adopted throughout to determine statistical significance.

## Results

### Expression of Oct4A in serous ovarian tumors

**Fig. 1:**
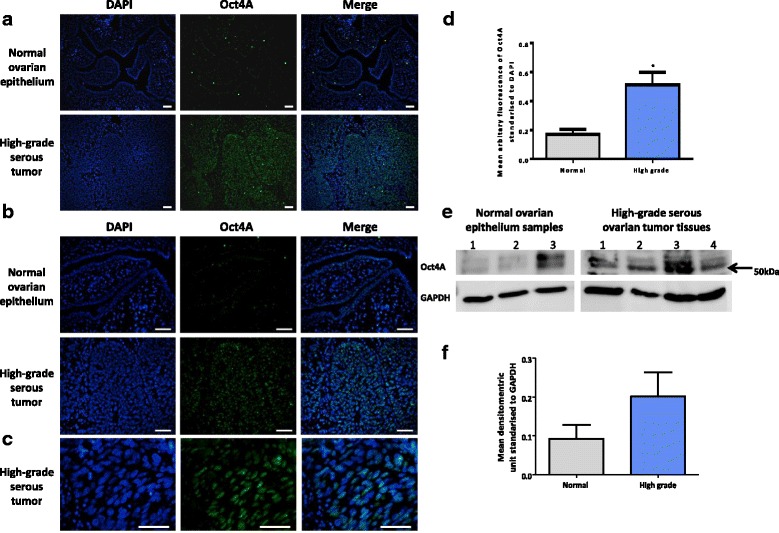
Expression and localization of Oct4A in normal ovarian epithelium tissues and high-grade serous ovarian tumors. **a**-**d** The expression and localisation of Oct4A in normal ovarian epithelium and primary grade 3 serous tumor samples was evaluated using immunofluorescence staining. Tumor tissues were immunostained using a mouse monoclonal Oct4A-specific antibody and visualized using the secondary Alexa 488 fluorescent labelled antibody (green). Nuclear staining was visualized using DAPI staining (blue). Images show Oct4A expression in the nuclei of tumor cells. Images are representative of *n* = 3 normal ovarian epithelium and *n* = 3 primary grade 3 serous ovarian tumor samples. Magnification is set at **a** 100x, **b** 200x and **c** 400x. Scale bar represents 100 μM. **d** Mean fluorescence intensity of Oct4A staining was quantified on images taken at 200x magnification using Cell-R software. Data is presented as the mean ± SEM of Oct4A expression standardized to DAPI of three independent samples. Significance is indicated by *P < 0.05 as determined by student’s *t*-test. **e** Nuclear cell lysates extracted from human normal ovarian epithelium tissues and primary high-grade serous ovarian tumors were evaluated for Oct4A protein expression using Western blot analysis. The Oct4A protein band of interest is indicated by an arrow at ~50 kDa. Total protein load was determined by stripping and re-probing the membrane with GAPDH. **f** Densitometry analysis of Oct4A Western blots. Data is expressed as a ratio of Oct4A protein expression standardized to GAPDH and presented as the mean ± SEM

We have previously shown enhanced expression of Oct4A in different histological grades of serous ovarian tumors compared to normal ovarian epithelium by immunohistochemistry [19]. In this study, we confirmed our previous results on normal and high-grade serous tumors using immunofluorescence and Western blot techniques. We demonstrate signifcant nuclear expression of Oct4A in high-grade serous tumors compared to normal ovarian tissues by immunofluorescence (Fig. 1a-d). This was confirmed by Western blot analysis performed on nuclear extracts of normal and tumor samples, where a higher Oct4A expression was observed in serous tumor samples compared to normal ovarian tissues (Fig. 1e-f). However due to small sample size, this result was not statistically significant (*P* = 0.226). These results confirm the nuclear localisation and expression of the stem cell specific Oct4A isoform in serous ovarian tumors and confirms that Oct4A expression increases in high-grade serous tumors compared to normal ovaries.

### Association between endogenous Oct4A expression and sphere forming abilities of ovarian cancer cell lines

The positive identification of Oct4A in primary serous ovarian tumors suggests a possible biological role of Oct4A in the disease. Using quantitative real-time PCR analysis, we have previously shown mRNA expression of Oct4A in four ovarian cancer cell lines OVCAR5, SKOV3, OVCA 433 and HEY [19]. All cell lines with the exception of OVCA433 expressed significantly elevated levels of Oct4A mRNA when compared to the immortalized normal ovarian surface epithelium cell line ISOE398 [19]. The HEY cell line was found to express the greatest endogenous level of Oct4A mRNA with a 6-fold increase in Oct4A expression, compared to the normal ISOE398 cell line. OVCAR5 and SKOV3 cells both exhibited a 4-fold increase in Oct4A mRNA expression compared to the normal ovarian cell line ISOE398 [19].

**Fig. 2:**
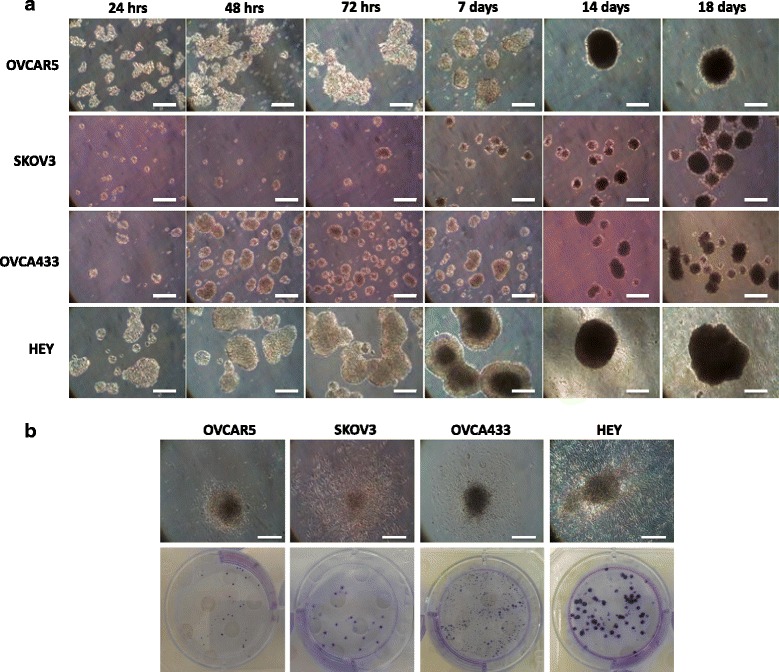
Sphere forming abilities and adhesion profiles of spheroids produced from epithelial ovarian cancer cell lines. **a** 1 x 10^5^ OVCAR5, SKOV3, OVCA433 and HEY cells were plated on ultra-low attachment plates in the presence of complete growth medium and sphere formation was monitored over 18 days. Images are representative of 3 independent experiments and taken from a section of a 6 well plate well using an inverted phase contrast microscope at 100x magnification. Scale bar represents 100 μM. **b** 18 day spheroids derived from OVCAR5, SKOV3, OVCA433 and HEY cells were collected, plated and cultured on plastic for a further 24 h. Images of adhered spheres were taken using an inverted phase contrast microscope at 100x magnification. Scale bar represents 100 μM. Adhered spheroids were also assessed by crystal violet staining to determine colony formation. Images are that of an entire well of a 6 well plate and representative of 3 experiments prepared in triplicate

As the formation of multi-cellular aggregates within the ascites of ovarian cancer patients has been described as an important feature for the long term preservation of CSCs [27, 28], and also indicative of advanced-stage aggressive disease [28, 29], the expression of Oct4A was correlated to the ability of each cell line to form non-adherent spheres in ultra-low attachment plate cultures over 18 days (Fig. 2a). Within 24 h of culture, all four ovarian cancer cell lines demonstrated the ability to form loose multicellular aggregates (Fig. 2a). However, with prolonged time in culture, loose aggregates eventually formed tightly compact, rounded spheres which increased in size over time (Fig. 2a). Detailed morphological analysis of spheres using light microscopy demonstrated HEY cells to be the first to produce dense compact spheres 7 days post plating. Conversely, OVCAR5, SKOV3 and OVCA433 cells all required a minimum of 14 days in culture to form compact spheres (Fig. 2a). By day 18 in culture, noticeable differences in sphere sizes were noted between the cell lines, with OVCA433 cells producing the smallest spheres in diameter (~50–100 μM) followed by SKOV3 (~75–150 μM), OVCAR5 (~100–250 μM) and HEY (200–500 μM).

The viability of 18 day spheres produced by the ovarian cancer cell lines was assessed by their capacity to adhere to plastic. Under light microscopy, the spheres produced by the ovarian cancer cell lines adhered to plastic within 24 h (Fig. 2b). Further microscopic evaluation demonstrated these adhered spheres underwent cellular dispersion away from the sphere core. This effect was predominately seen in spheres produced by Oct4A abundant HEY cells and to a lesser extent in spheres derived from other ovarian cancer cell lines. The expression of Oct4A however, was not related to the degree of adhesion of the spheres, with Oct4A low expressing OVCA433 producing several smaller adhered colonies compared to those produced by high Oct4A expressing HEY cell line (Fig. 2b). OVCAR5 and SKOV3 cell lines which had moderate endogenous level of Oct4A produced several colonies which were both smaller and fewer when compared to those produced by HEY cells.

### shRNA mediated knockdown of Oct4A in HEY ovarian cancer cell line

**Fig. 3:**
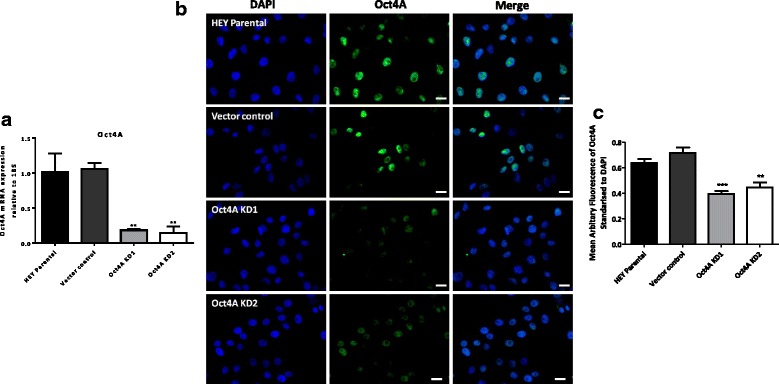
Stable shRNA knockdown of Oct4A in HEY ovarian cancer cell line. **a** The efficiency of Oct4A knockdown was evaluated by quantitative real-time PCR analysis. Relative quantification of Oct4A expression was standarized to 18S housekeeping gene and normalised to vector control cells. Data is from three independent samples assessed in triplicate. **b** The expression and localization of Oct4A in HEY cells following stable shRNA knockdown of Oct4A was also evaluated using immunofluorescence staining. Cells were immunostained using mouse monoclonal Oct4A-specific antibody and visualized using the secondary Alexa 488 fluorescent labelled antibody (green). Nuclear staining was visualized using DAPI staining (blue). Images are representative of three independent experiments. Magnification is set at 200x. Scale bar is set at 50 μM. **c** Mean fluorescence intensity of Oct4A staining is presented as the mean ± SEM of Oct4A expression standardized to DAPI of three independent experiments. Data is from three independent samples assessed in triplicate. Significance is indicated by **P < 0.01 and ***P < 0.001 as determined by One-Way ANOVA using Dunnett’s Multiple Comparison post-test against the HEY vector control

In order to determine the potential biological functions of Oct4A in serous ovarian tumors, stable Oct4A knockdown clones were generated in the metastatic ovarian cancer cell line HEY using shRNA-mediated methods as described previously [19]. Two clones Oct4A KD1 and Oct4A KD2 which showed a knockdown efficiency of ~80 % at the mRNA level (Fig. 3a) and a significant 50–60 % decrease at the protein level compared to vector control cells (Fig. 3b-c) was used in this study.

### Diminution of Oct4A suppresses integrin β1 and α5 subunits expression in HEY spheres

**Fig. 4:**
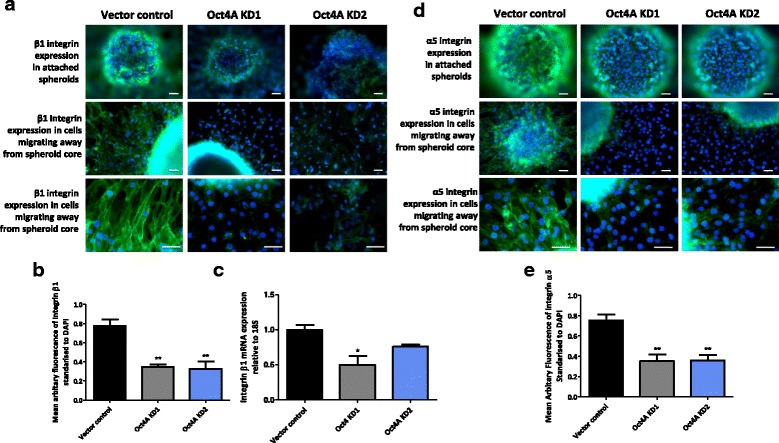
β1 and α5 integrin subunit expression and localisation in HEY Oct4A knockdown spheres. Expression and localisation of β1 and α5 integrin subunits in 18 day Oct4A KD spheres was evaluated using immunofluorescence staining. Adhered spheres were immunostained using mouse monoclonal antibodies for integrin **a** β1 or integrin **d** α5 and visualised using the secondary Alexa 488 fluorescent labelled antibody (green). Nuclear staining was visualised using DAPI staining (blue). Magnification is set at 100x for top and middle image panels and 200x for images in the bottom panel. Scale bar is set at 50 μM. Mean fluorescence intensity of **b** β1 and **e** α5 staining is presented as the mean ± SEM of respective integrin expression standardised to DAPI from 3 independent experiments. **c** Quantitative real-time PCR analysis was used to determine the expression of integrin β1 subunit in non-adherent 18 day Oct4A KD spheres. Relative quantification of mRNA expression was standardised to 18S housekeeping gene and normalised to the vector control. Data is expressed as the mean fold change ± SEM from three independent samples assessed in triplicate. Significance is indicated by *P < 0.05 and **P < 0.01 as determined by One-Way ANOVA using Dunnett’s Multiple Comparisons post test against the vector control

We have previously shown that suppression of Oct4A in HEY cells resulted in an inability of HEY cells to form tightly compact spheres as well as subsequent adherence on plastic [19]. We and others have previously shown that integrins can mediate several aspects of ovarian tumor cell behavior including tumor cell matrix adhesion, sphere formation and peritoneal metastasis [15, 30, 31]. In this study we investigated the profile of the integrin β1 subunit in adhered Oct4A KD spheres after 18 days in culture by immunofluorescence. As expected, integrin β1 expression was localized in the membrane of cells making up the spheres as well as in cells migrating away from the sphere cores (Fig. 4a). When assessed semi quantitatively, the expression of the integrin β1 subunit was significantly reduced ~50 % in the cells migrating away from the sphere core of adhered Oct4A KD spheres compared to vector control spheres (Fig. 4b). The results of quantitative real-time PCR analysis correlated with immunofluorescence analysis, with Oct4A KD1 non-adherent spheres demonstrating a significant 50 % decrease in integrin β1 subunit mRNA expression compared to non-adherent vector control spheres (Fig. 4c). Although not significant, Oct4A KD2 non-adherent spheres demonstrated a 30 % decrease in integrin β1 mRNA expression compared to non-adherent vector control spheres.

Given that the integrin β1subunit is known to couple with several α integrin sub-units, the expression of the integrin α5 subunit was also investigated in adhered Oct4A KD spheres using immunofluorescence analysis (Fig. 4d and e). Similar, to that of integrin β1, the integrin α5 subunit was significantly down regulated by 50 % in both Oct4A KD1 and Oct4A KD2 spheres compared to vector control spheres. The localization of integrin α5 remained confined to the cell membranes making up the spheres and migrating cells.

### Attenuation of Oct4A suppresses integrin β1, α2 and α5 subunit expression in HEY monolayer cells and affects the adhesive ability of HEY cells to ECM proteins collagen and fibronectin

**Fig. 5:**
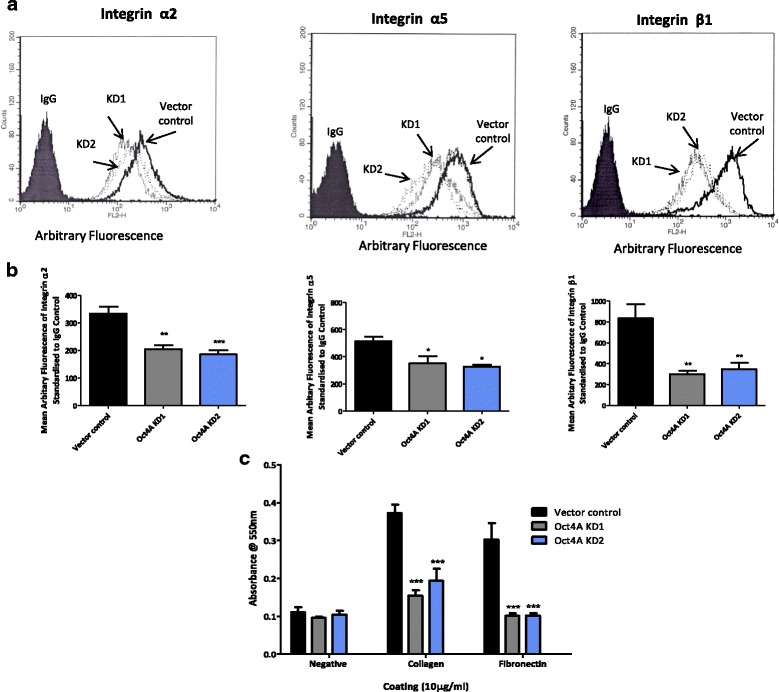
Expression of integrin α2, α5 and β1 subunits in HEY Oct4A knockdown monolayer cells as determined by flow cytometry. **a** Flow cytometric analysis was used to examine integrin α2, α5 and β1 subunit expression in monolayer Oct4A KD cells. Cells were cultured, collected and incubated with respective primary antibodies or negative IgG control before being detected using a secondary goat anti-mouse IgG conjugated with phycoerythrin. Vector control cells are represented by solid black peaks and broken peaks are indicative of Oct4A KD cells as indicated. The filled histogram denotes the negative control IgG. **b** Semi-quantitative analysis of flow cytometry results. Results are expressed as the difference between the arbitrary fluorescence expression of the integrin of interest and the negative control IgG and presented as the mean ± SEM of four independent experiments (except integrin α5, *n* = 3). Significance is indicated by *P < 0.05, **P < 0.01 and ***P < 0.001 as determined by One-Way ANOVA with Dunnett’s Multiple Comparison post-test compared to vector control. **c** The adhesive ability of Oct4A KD cells on collagen and fibronectin was determined by 24 h adhesion assay. The number of adhered cells was estimated by measuring the optical absorbance at OD_550nm_ with the SpectraMax190 Absorbance Microplate Reader. Data is expressed at the mean ± SEM of three independent experiments performed in triplicate. Significance between Oct4A KD cells and vector control cells per ECM group is indicated by ***P < 0.001 as determined by Two-Way ANOVA and Bonferroni post-test

The cell surface expression of integrin β1 and associated α-subunits was further assessed in monolayer cultures of Oct4A KD cells by flow cytometry (Fig. 5a and b). The results revealed the expression of β1, α2, and α5 integrin subunits to be significantly down regulated in HEY cells following shRNA-mediated knockdown of Oct4A compared to vector control cells. Endogenous expression of integrin β1 was seen to decrease ~65–70 %, while integrin α2 expression was suppressed 30–40 %. α5 was seen to decrease by 30–40 % (Fig. 5a-b). Loss of β1, α2, α5 integrin subunits also correlated with a significant decrease in cellular adhesion to collagen and fibronectin by Oct4A KD cells compared to vector controls (Fig. 5c).

### Diminution of Oct4A reduces secreted levels of pro-MMP2 but not MMP9 in monolayer cultures of HEY cells

**Fig. 6:**
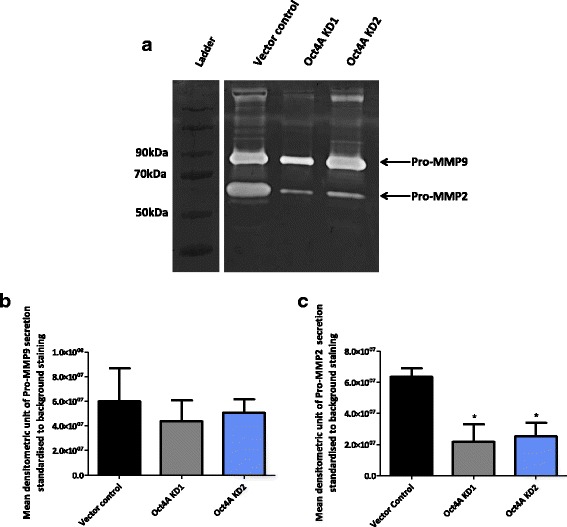
MMP9 and MMP2 secretion by HEY Oct4A KD monolayer cells. **a** Gelatin zymography analysis of MMP9 and MMP2 secretion by HEY Oct4A KD cells was performed on a 10 % polyacrlamide gel containing 1 % gelatine. Pro-MMP9 (~92 kDa) and Pro-MMP2 (~72 kDa) gelatinolytic activity is indicated by clear banding. **b**-**c** Densitometric analysis of gelatin zymography was performed to determine secreted Pro-MMP9 and Pro-MMP2 activity. Enzymatic activity is expressed as the intensity of the Pro-MMP9 and Pro-MMP2 bands of interest and presented as the mean ± SEM from three independent experiments. Significance is indicated by *P < 0.05 as determined by One-Way ANOVA using Dunnett’s Multiple Comparison post-test against HEY vector control cells

Successful metastasis of disseminated ovarian cancer cells is not only driven by tumor cell adhesion in the peritoneal cavity, but also requires active degradation of extracellular matrix (ECM) to invade the mesothelial lining of the peritoneum [32, 33]. MMP secretion and activity by ovarian tumor cells have previously been implicated in the remodeling of ECM during the process of metastasis [34]. As a measure of invasive ability, the secretion of pro-MMP2 and pro-MMP-9 by Oct4A KD cells was investigated using gelatin zymography (Fig. 6). Pro-MMP2 levels were significantly reduced by ~70 % in the conditioned medium obtained from Oct4A KD cells compared to vector control cells (Fig. 6a-b). However, no significant change in pro-MMP9 secretion was observed when compared to vector control cells (Fig. 6c). Activated secretion of MMP2 and MMP9 could not be detected.

### Attenuation of Oct4A expression decreases in vivo tumorigenicity of HEY cells in the BALB/c nude mouse model

**Fig. 7:**
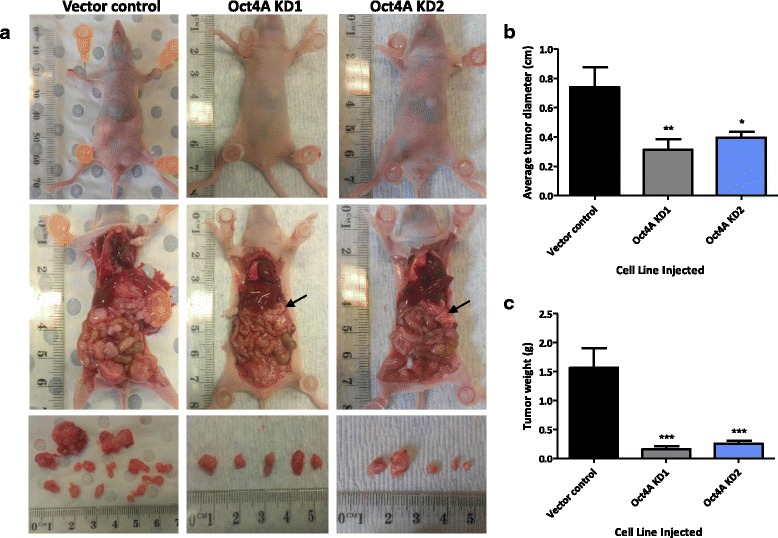
Tumor development by HEY Oct4A knockdown cells in BALB/c nude mice. **a** HEY vector control, Oct4A KD1 or Oct4A KD2 cells were inoculated by ip injection into 6–8 week old female BALB/c nude mice (*n* = 8 group). Mice were monitored daily for tumor development before being culled, dissected and photographed at 4 weeks post injection. Arrows indicate the location of small tumor nodules. **b** Average tumor size debulked from mice inoculated with vector control and Oct4A KD cells. Each tumor excised was individually measured before tumour size averaged for each mouse. Data is expressed as the mean ± SEM of tumour size (cm) for each group (*n* = 8 mice/group). **c** Average weight of tumors derived from vector control and Oct4A KD1 and Oct4A KD2 mice. Data is presented as the mean ± SEM of tumor weight (grams) obtained from each mouse (*n* = 8 mice/group)

To investigate the biological relevance of Oct4A in vivo, intraperitoneal (ip) xenografts were developed with Oct4A KD cells and vector control cells in Balb/c nude mice. Four weeks post inoculation, 90 % of mice (10/11) injected with vector control cells displayed characteristics of metastatic advanced-stage disease including abdominal distention and weight loss (Fig. 7). In comparison, mice injected with Oct4A KD cells appeared to be healthy with no apparent visible tumors within 4 weeks post inoculation. However, after surgical dissection, there were tumors which were fewer in number and significantly smaller in size and weight compared to mice injected with vector control cells (Fig. 7a-c). On average, mice injected with vector control cells produced tumors which were 7.4 mm ± 1.4 in diameter. In comparison, tumors derived from Oct4A KD cells were approximately 50 % smaller in size. Tumors derived from Oct4A KD1 cells were 3.1 mm ± 0.7 in diameter, while those derived from Oct4A KD2 cells produced tumors 3.9 mm ± 0.40 in diameter (Fig. 7b). A similar trend was observed when considering total tumor weight, with mice injected with vector control cells developing an average l tumor weight of 1.60 g ± 0.34, equivalent to 12.58 % ± 1.88 of total mouse body weight (Fig. 7c). In contrast, tumors derived from mice inoculated with Oct4A KD1 cells exhibited an average tumor weight of 0.15 g ± 0.05, equivalent to a tumor burden of 1.33 % ± 0.45 of total mouse body weight, while Oct4A KD2 mice exhibited a tumor weight of 0.25 g ± 0.04, equivalent to 2.33 % ± 0.41 of total body weight (Fig. 7c). This indicates that suppression of Oct4A in HEY cells reduces the formation of tumors in vivo by 80–90 % compared to vector control cells within the first few weeks of tumor development.

### Knockdown of Oct4A reduces the expression of CK7, Glut-1, CD31 and CD34 in xenografts derived from Oct4A KD cells compared to vector control cells

**Fig. 8:**
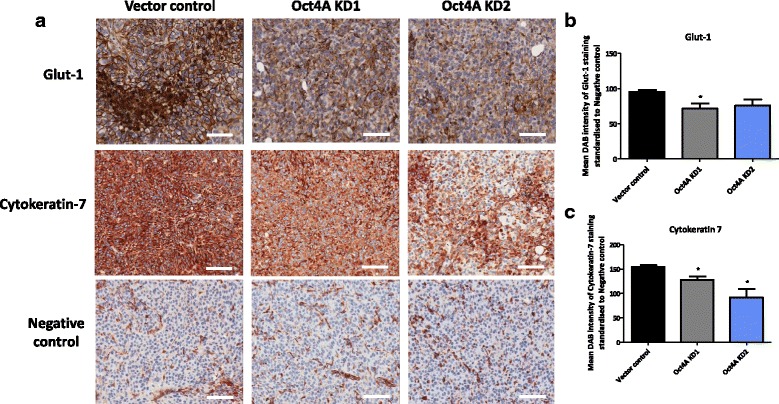
Immunohistochemical analysis of Glut-1 and CK7 expression in Oct4A knockdown xenografts. **a** Representative immunohistochemistry staining images of debulked mouse xenografts for the expression of Glut-1 and CK7. Images are set at 200x magnification and scale bar represents 50 μM. **b**-**c** Quantification of Glut-1 and CK7 staining was determined by using Image J software recognizing DAB intensity. Variations in staining were determined by subtracting the negative control DAB reading from the DAB reading of the protein of interest for each xenograft. Data is presented as the mean ± SEM of staining intensity (*n* = 4/group). Significant variations between Oct4A KD groups and vector control was determined by Student’s *t*-test *P < 0.05

Immunohistochemical analysis of mouse tumors revealed that the expression of CK7, an important marker used for the diagnosis of ovarian cancer [34], was significantly reduced by 1.1–1.6 fold in tumors derived from Oct4A KD cells (Fig. 8a-b), compared to tumors derived from vector control cells. Significant reduction in CK7 in Oct4A KD derived tumors correlated with ~30 % reduction in the expression of glucose transporter Glut-1 in tumor xenografts (Fig. 8a-b). The staining of CK7 and Glut-1 was predominately localized to the cytoskeletal membranes of tumor cells. However, reduced expression of Glut-1 was only significant in tumor xenografts derived from Oct4A KD2 cells.

**Fig. 9:**
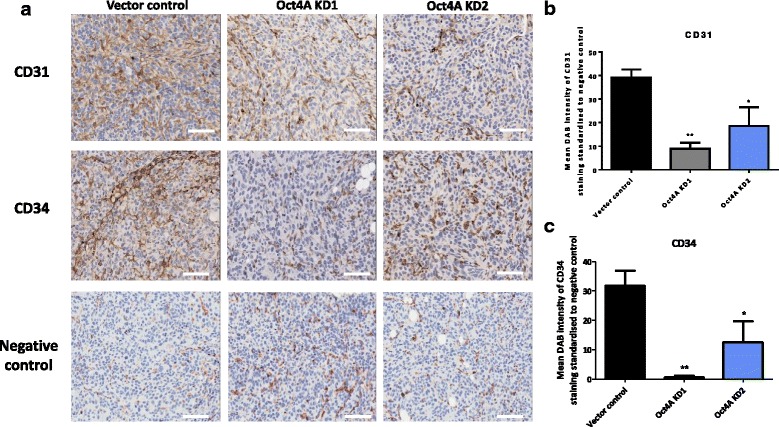
Immunohistochemical analysis of CD31 and CD34 expression in Oct4A knockdown xenografts. **a** Representative images of immunohistochemistry staining of debulked mouse xenograft tumors for the expression of CD31 and CD34. Images are set at 200x magnification and scale bar represents 50 μM. **b**-**c** Quantification of CD31 and CD34 staining was determined as described in Figure 9. Significant variations between Oct4A KD groups and vector control was determined by One-Way ANOVA using Dunnett’s Multiple Comparison post-test **P < 0.01 and *P < 0.05

To assess whether the reduced tumor size/weight in Oct4A KD cell-derived tumors may result from reduced tumor vascularity, we assessed the expression of human specific angiogenesis markers CD31 and CD34 on mouse xenografts. Compared to tumors derived from vector control cells, the staining intensity of both CD31 and CD34 were significantly reduced in tumors derived from both Oct4A KD cell lines (Fig. 9). Specifically, CD31 expression was reduced 4-fold and 2-fold in tumors derived from Oct4A KD1 and Oct4A KD2 cells respectively. Similarly, CD34 expression was reduced by 16-fold in tumor xenografts derived from Oct4A KD1 cells compared to only 2.5-fold decrease in Oct4A KD2 cells derived xenografts. These observations were consistent with the reduced tumor size, weight and growth potential observed in tumor xenografts derived from Oct4A KD compared to vector control cells.

## Discussion

It is well recognized that the interaction between tumor cells and the ECM in the rapidly evolving tumor microenvironment is essential for the generation of regulatory signals that ultimately determine the fate of tumor cells and influence the evolution of a malignant phenotype [35]. The integrin family of cell surface receptors is an important component of the ECM which sense micro environmental changes and trigger a range of cellular responses by forming a physical link between the inside and outside of tumor cells. Moreover, they allow for the bidirectional regulation of signals necessary to promote tumor progression [14]. However, it is not yet clear if the effects that integrins have in modulating tumor cell behavior are also related to their associated role with CSCs in the tumor microenvironment. In this study we demonstrate a direct association between CSC specific Oct4A transcription factor expression and the β1 family of integrins primarily the α2 and α5 subunits.

In a previous study, we demonstrated significantly enhanced expression of the stem cell specific Oct4A isoform which increased according to histological grades of serous ovarian tumors compared to normal ovarian epithelium by immunohistochemical methods [19]. In this study we further validate these results in high-grade serous ovarian tumor samples using immunofluorescence and Western blot techniques. We demonstrate that the expression of Oct4A is enhanced in high-grade serous samples compared to normal ovaries and localized to the nucleus of tumor cells. These results support the hypothesis that Oct4A has a specific role in ovarian cancer progression.

We have previously demonstrated that suppression of Oct4A is capable of inhibiting sphere forming abilities in the HEY ovarian cancer cell line [19]. To expand on this observation, the expression of Oct4A in a range of ovarian cancer cell lines was seen to be directly correlated to the anchorage independent sphere forming abilities of each cell line. Sphere formation has been identified as a property of CSCs and the phenomenon has been reported in leukemia and in solid tumors of breast, colon and brain [27]. However, the molecular mechanisms of how sphere-forming cells retain their stem-like characteristics remain unknown. Recent studies, have demonstrated that mouse ESCs are capable of maintaining long-term stemness within a 3D scaffold by manipulating integrin signaling [36, 37]. These studies reported greatly increased expression of known stem cell markers Oct4 and Nanog was associated with simultaneous activation of Akt1 and Smad1/5/8 by α5β1, αvβ5, α6β1 and α9β1 integrins within the 3D scaffold. This maintained the self-renewal capacity of ESCs in the absence of leukemia inhibitory factor (LIF) signaling [37]. These studies suggest that a ‘stem cell niche-specific integrin signaling mechanism’ within a 3D microenvironment can sustain the survival of ESCs without signals received from growth factors like LIF which are absolutely essential for the survival of ESCs.

Many of the same integrins which support ESC fate are also markers of CSCs in different cancers [15]. These include α6 integrin enriching for CSCs in breast [38], prostate [39], squamous cell carcinoma [40] and colorectal cancer [41]. Integrin β3 is critical for stemness in breast [42, 43], pancreas [44] and lung cancer [44], while β1 integrin is necessary for the stemness characteristics in glioblastomas [45]. These studies are in agreement with our findings which demonstrate that integrins β1, α2 and α5 were down regulated by the knockdown of stem cell-specific Oct4A expression in HEY cell line. These observations were made in cells maintained as monolayer as well as sphere cultures and were consistent with the loss of adhesion of these cells on collagen and fibronectin. However, it should be noted, that integrins can also influence CSC niches independent of their capacity to interact with ECM [15].

We have previously shown that enhanced expression of α2β1 integrin in ovarian cancer spheres facilitates sphere disaggregation, pro-MMP-2/9 expression and MMP-2/9 activation [31]. In the current study, this is consistent with a decrease in α2 and β1 integrin expressions in Oct4A knockdown HEY cells along with reduced pro-MMP-2 secretion. This data correlates to the loss of sphere disaggregating and migratory ability previously shown in Oct4 KD cells [19]. These observations in our Oct4A knockdown model are consistent with observations in pancreatic cell line models, where loss of Oct4 also resulted in reduced MMP2 expression and subsequent reduced tumor cell invasive ability [46]. Interestingly, MMP2 is known to actively degrade fibronectin and collagen I [32, 33, 47], thus potentially linking these results to the decreased ability of Oct4A KD cells to adhere to fibronectin and collagen I. In the pancreatic cancer cell line model, diminution of Oct4 was associated with a decrease in MMP9 expression [48]. In our study, no such relationship in Oct4A KD cells was observed. Interestingly however, the Oct4B isoform has been identified to regulate both MMP2 and MMP9 expression in cervical cancer, suggesting the lack of MMP9 suppression may be due to the fact that Oct4B expression was not specifically suppressed in the current study [49].

We also demonstrate that the suppression of Oct4A in HEY cells resulted in a significant reduction in the tumor growth, weight and size in Balb/c nude mouse model. In addition, tumor xenografts derived from Oct4A KD cells displayed relatively lower abundance of markers associated with ovarian cancer (CK7), cancer metabolism (Glut-1) and angiogenesis (CD31 and CD34). This indicates that the suppression of Oct4A not only reduced the tumor initiating ability of cells in vivo but also resulted in a reduction in angiogenic potential which consequently may have resulted in slowed or abrogated tumor growth. These results are consistent with the positive correlation between the expression of Oct4 and vasculogenic mimicry formation and poor prognosis in breast cancer patients [50]. In addition, Oct4 has been shown to promote glioblastoma progression through vascular endothelial growth factor production [51]. Importantly, integrins are well documented to play significant roles in mediating tumor vascularity and angiogenesis [52]. Specifically, integrin α5β1 has been identified to play a role in tumor angiogenesis in in vivo mouse models [53, 54]. Collectively, this tie in with the reduced expression of β1 and α5 subunits demonstrated in Oct4A KD cells and overall reduced tumorigenic and angiogenic profiles in this study.

## Conclusions

**Fig. 10:**
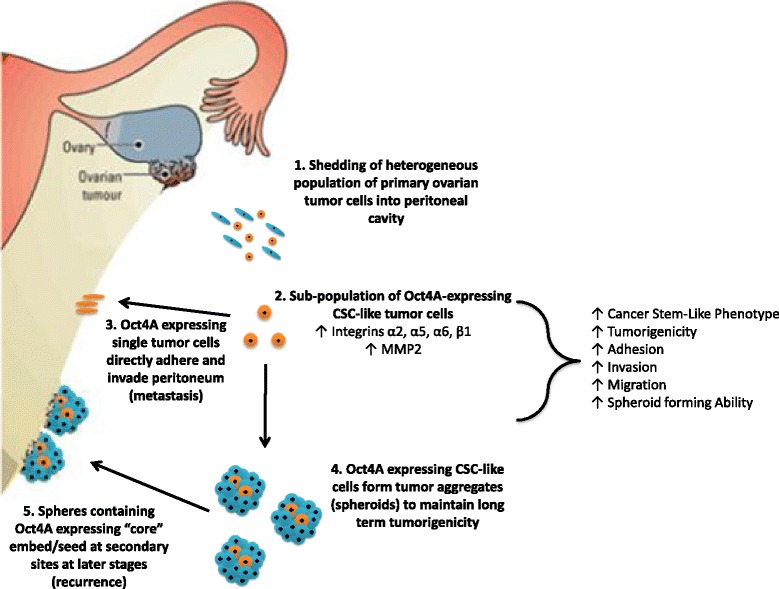
Proposed model of integrin mediated ovarian cancer metastasis regulated by Oct4A. A population of Oct4A-expressing tumor cells which disseminate directly into the peritoneal cavity are capable of surviving traditional combination therapy consisting of cisplatin (or carboplatin) and paclitaxel. These cells are able to regulate the expression of a range of integrin cell surface receptors. This promotes the formation of multicellular tumor aggregates (spheres) and assists tumor cells to directly adhere, invade and migrate at a metastatic site thus initiating secondary/recurrent disease

In summary, while Oct4 has previously been shown to regulate several processes involved in solid tumor metastasis, the role of the Oct4A isoform in ovarian cancer progression is still emerging. The results of this study demonstrate a crucial role for the pluripotent stem cell specific Oct4A isoform in regulating key events required for ovarian cancer progression, survival and metastasis. Importantly, these results were associated with altered expression of α2, α5 and β1 integrin sub-units. This study has therefore revealed a complex novel relationship between Oct4A and cell surface membrane receptors which may potentially drive the events involved in ECM remodeling which are crucial for ovarian cancer metastasis in the peritoneal microenvironment. From these results, it could be hypothesized that following exfoliation into the peritoneal cavity, Oct4A expressing ovarian tumor cells are capable of regulating the expression of a specific sub-set of cell surface integrin receptors. This in turn would assist in the formation of non-adherent spheres within the ascites fluid of advanced stage patients thus driving long-term tumorigenicity. Moreover, through its regulation of integrin expression, Oct4A would be able to drive cellular adhesion, invasion and tumor survival thus promoting ongoing metastasis (Fig. 10). Overall, the results of this study collectively suggest targeting Oct4A through novel therapeutics may help overcome ovarian cancer peritoneal metastasis.

## Abbreviations

ANOVA, analysis of variance; CK7, cytokeratin 7; CSCs, cancer stem cells; DAPI, 4’,6-diamidino-2-phenylindole; ECM, extracellular matrix; ESC, embryonic stem cells; IP, intraperitoneal; iPSCs, induced pluripotent stem cells; LIF, leukaemia inhibitory factor; MMP, matrix metalloproteinase; SDS-PAGE, sodium dodecyl sulphate polyacrylamide gel electrophoresis
